# Skin Pigmentation in Gilthead Seabream (*Sparus aurata* L.) Fed Conventional and Novel Protein Sources in Diets Deprived of Fish Meal

**DOI:** 10.3390/ani10112138

**Published:** 2020-11-17

**Authors:** Domitilla Pulcini, Fabrizio Capoccioni, Simone Franceschini, Marco Martinoli, Emilio Tibaldi

**Affiliations:** 1 Consiglio per la Ricerca in Agricoltura e l’Analisi dell’Economia Agraria, Centro di Ricerca di Zootecnia e Acquacoltura, Via Salaria 31, Monterotondo, 00015 Rome, Italy; domitilla.pulcini@crea.gov.it (D.P.); marco.martinoli@crea.gov.it (M.M.); 2 Laboratory of Experimental Ecology and Aquaculture, Department of Biology, University of Rome “Tor Vergata”, Via della Ricerca Scientifica, 00133 Rome, Italy; Simone.Franceschini@uniroma2.it; 3 Dipartimento di Scienze Agroalimentari, Ambientali e Animali, Università di Udine, Via delle Scienze 206, 33100 Udine, Italy; emilio.tibaldi@uniud.it

**Keywords:** carotenoids, crayfish, gilthead seabream, image analysis, insects, microalgae, pigmentation, poultry by-products, vegetable protein sources

## Abstract

**Simple Summary:**

Intensive farming of carnivorous fish species relies on the use of feeds, where fish meals have represented, for a long while, ideal sources of protein to ensure optimal growth, health and quality to cultured fish. Due to the gap between the demand for these commodities by the growing aquaculture industry and the unsustainability of further exploitation of the alieutic resources, their levels of inclusion in fish feeds have been mostly replaced by terrestrial plant protein-rich derivatives and conventional animal processed proteins. Recently, novel ingredients (i.e., insects and microalgae) have been proposed to this end. While the impact of different alternative proteins on fish growth and health has been studied, limited information exists on the effects of such dietary changes on quality traits of cultured fish such as skin pigmentation. The present study was aimed at assessing the pattern of yellow pigmentation of the skin in gilthead seabream fed various alternative protein sources (vegetable ingredients, insects, poultry by-product meal, red swamp crayfish and marine microalgae) included in different proportions in fishmeal-free diets, in order to evaluate new feed formulations on the basis of their coloring capacity, as intense skin coloration have been associated with high-quality of farmed fish products.

**Abstract:**

The pattern of yellowish pigmentation of the skin was assessed in gilthead seabream (*Sparus aurata*) fed for 12 weeks iso-proteic (45%) and iso-lipidic (20%) diets deprived of fish meal and containing either a blend of vegetable protein-rich ingredients or where graded levels of the vegetable protein blend were replaced by insect (*Hermetia illucens*—10%, 20% or 40%*)* pupae meal, poultry by-product meal (20%, 30% or 40%), red swamp crayfish meal (10%) and marine microalgae (*Tisochrysis lutea* and *Tetraselmis suecica*—10%) dried biomass. Digital images of fish fed diets differing in protein sources were analyzed by means of an automatic and non-invasive image analysis tool, in order to determine the number of yellow pixels and their dispersion on the frontal and lateral sides of the fish. The relationship between the total carotenoid concentration in the diet and the number of yellow pixels was investigated. Test diets differently affected gilthead seabream skin pigmentation both in the forefront and the operculum, due to their carotenoid content. The highest yellow pixels’ number was observed with the diet containing microalgae. Fish fed poultry by-product meal were characterized by the lowest yellow pixels’ number, diets containing insect meal had an intermediate coloring capacity. The vegetable control, the microalgae mix diet and the crayfish diet had significantly higher values of yellow pixels at both inspected skin sites.

## 1. Introduction

Farmed fish external traits, such as skin or muscle color and body shape, are increasingly important for consumers’ acceptance of aquatic food products [1,2]. Producers have to manage these traits ever more closely, but this represents a big challenge, as color and shape are very complex traits, with a complex genetic architecture and under the influence of several environmental factors. As an example, undesirable dark coloration of farmed fish respect to their wild counterparts is often due to an excessive production of melanin in response to stressful farming conditions and protein-rich diets [3]. For certain species, such as red porgy *Pagrus pagrus* or red sea bream *Pagrus major*, a natural red skin pigmentation similar of that of wild specimens is pivotal from a commercial point of view, increasing consumer acceptance of aquatic food [4] and, thus, allowing an increase in market price. Farmed red porgy exhibit a dark-grey skin pigmentation if fed diets not supplemented with astaxanthin, and this often leads to rejection by the consumers [5]. Final products often gain added value on the market if skin pigmentation shows certain attributes: more intense flesh and skin coloration has been traditionally associated with superior and more natural taste and with healthy aquatic products [6,7,8,9,10,11]. This is particularly true for species that are commercialized in their entirety. Furthermore, as carotenoids are demonstrated to have beneficial effects, skin or muscle red coloration is considered to be an index of animal welfare in farmed fish [12]. Red and yellow hues, given by the storage of carotenoids in integument or muscle, are the most important for commercial fish species, such as Atlantic salmon, rainbow trout, red porgy and gilthead seabream. Fish are not able to synthesize carotenoids, but they can modify and store pigments present in the diet. Diets for farmed fish are enriched with different carotenoids (i.e., synthetic as astaxanthin and cantaxanthin, or natural as pigment from algae, red yeast or crustaceans), in order to maintain a skin pigmentation as natural as possible, enhance growth and performance of broodstock, and provide protection against oxidation. 

Wild gilthead seabream *Sparus aurata* L. is characterized by golden yellow coloration on its forefront and operculum. Farmed fish often exhibited darker gray or fade pigmentation [13,14,15,16]. Supplementation of artificial diet with carotenoids has been demonstrated to enhance skin pigmentation in gilthead seabream [17,18,19], even if other environmental factors and stressors due to aquaculture conditions must be considered. 

The available knowledge of the effects of diet on skin pigmentation in Sparidae is reported in Table 1.

Previous studies were mainly focused on the effect on skin pigmentation of diet supplemented with synthetic carotenoids [5,10,17,18,22] or with natural functional ingredients rich in pigments (i.e., microalgae biomasses [18], or crustacean meals [5,23,25]). The increasing consumer demand for natural products makes synthetic pigments ever less desirable [27,28,29], despite the production of these latter had been demonstrated to be cheaper [30] and environmentally sustainable [31]. Moreover, synthetic pigments have been demonstrated to have possible adverse effects on human health, and thus have been banned in many countries [32]. From a functional point of view, natural carotenoids are more valuable than the synthetic alternatives, as the different chemical structure of these latter might influence several properties related to biological function, as the antioxidant potential or the shelf life [33,34]. For the above reasons, the global carotenoids market is continuously growing, with a compound annual growth rate of 5.7% [35]. Furthermore, synthetic pigments are not admitted in organic production (Reg. EU 2018/848); thus, finding natural and available sources of pigments represent a challenge for aquafeed commodities.

Modern and sustainable fish feed formulations are developing to minimize the inclusion of fishmeal and optimize the use of conventional vegetable protein-rich ingredients and novel ingredients such as insect meals, microalgae and processed animal proteins [36]. While the impact of different dietary alternative proteins on fish growth and health has been and is currently extensively studied, limited information exists on the effects of such dietary changes on quality traits of cultured fish such as skin pigmentation, an attribute which deserves commercial interest in the case of gilthead seabream. To the best of our knowledge, the skin coloring capacity of protein-rich vegetable ingredients, or alternative novel dietary proteins (i.e., insect meal) which contain carotenoids and are and or will increasingly be used in fish feeds, has not yet been investigated in feeding this fish species. The goal of this study was to assess and compare the pattern of yellowish pigmentation of the skin in gilthead seabream fed diets deprived of fishmeal and containing either a blend of vegetable protein-rich ingredients as major protein sources or where graded levels of the vegetable protein blend were replaced by *Hermetia illucens* pupae meal, poultry by-product meal, red swamp crayfish meal and dried biomass of two marine microalgae (*Tisochrysis lutea* and *Tetraselmis suecica*). To this end, a specific automatic, repeatable and non–invasive image analysis tool was developed and used to quantify yellow pixels in a digital image.

## 2. Materials and Methods

Nine test diets were formulated to be grossly iso-proteic (45%), iso-lipidic (20%) and isoenergetic (22 MJ kg^−1^). The ingredient composition and proximate analysis of the test diets are shown in Table 2. A control feed rich in plant-derived ingredients (CV—vegetable control) was designed in order to obtain a 90:10 weight ratio between vegetable and marine protein as well as a 67:33 weight ratio between vegetable and fish lipid calculated from the crude protein and crude lipid contribution to the whole diet of all marine and plant-based dietary ingredients. The remaining diets, denoted as H10, H20, H40, P20, P40, H10P30, RC10 and MA10, were prepared by replacing graded levels (10%, 20% or 40%) of crude protein from the mixture of vegetable protein sources of the CV diet, by crude protein from a commercial defatted *Hermetia illucens* pupae meal (H), poultry by-product meal (P) singly or in combination, red swamp crayfish meal (RC) and of a blend (2:1 w:w) of the dried biomass of two marine microalgae (MA—*Tisochrysis lutea* and *Tetraselmis suecica*), while maintaining a same 67:33 vegetable to fish lipid ratio as in the CV diet. All diets were manufactured by SPAROS Lda (Portugal) by extrusion in two pellet sizes (3 and 5 mm) and stored at room temperature, in a cool and aerated room until used.

The feeding trial was carried out at the aquaculture facilities of the Department of Agrifood, Environmental and Animal Sciences of the University of Udine. It used 486 gilthead seabream juveniles selected to be uniform in size, from a resident stock of 1200 specimens. Fish were randomly allocated to 27 groups, each consisting of 18 individuals kept in an indoor marine re-circulating aquaculture system including twenty-seven 250-L tanks. The system was kept under constant day length and intensity (12 h per day at 400 Lux) by supplemental fluorescent light tubes. It also ensured nearly constant and optimal water quality conditions to sea bream (T 23.4 ± 0.75 °C; Salinity 31ppt; pH 8.0 ± 0.09; DO 5.9 ± 0.22 mgL^−1^; TAN < 0.02mgL^−1^; N-NO2 < 1.00 mgL^−1^). After stocking, fish were adapted over 2 weeks to the experimental conditions. At the end of this period, fish attained an average size of 49 ± 0.4 g and the 27 groups were assigned to the nine dietary treatments according to a random design with triplicate groups (tanks) per treatment. Fish were hand-fed the experimental diets over 12 weeks in two daily meals (8:00 am and 4:00 pm) until the first feed item was refused.

At the end of the trial, all fish were anesthetized with MS-22 and final weight and total length were measured. Total length was measured with an ichthyometer. Each fish was photographed in the left lateral and frontal sides with a high-resolution camera (13 real MP) mounted on a tripod. Fish were illuminated with a photographic lamp (illuminating power 6000 K). The focal distance between the camera and the fish was 40 cm for lateral side and 20 cm for frontal one. Color calibration was carried out with a Color Checker 24 color patch. Background was eliminated from each image using Photoshop CS6. The RGB range of yellow was determined randomly sampling five yellow pixels in three images for each tank (9 for treatment), to define upper and lower values for red, green and blue values. The obtained RGB range was: R = 205–255, G = 130–255 and B = 000–015. These values were perturbed by ± 5%, but the results did not change.

For each processed image (n = 481), the following were determined: (1) the total number of yellow pixels in the defined RGB range, and (2) the Standard Distance Deviation (SDD), computed as the standard deviation of the mean distance among yellow pixels. SDD can be considered as a dispersion index of yellow pixels on the image: the higher is the value, the larger is the yellow pixels scattering along the profile. All data were collected with “countcolors” v. 0.9.1 R package (available at https://cran.r-project.org/web/packages/countcolors/vignettes/Introduction.html). Yellow pixels, once identified, were plotted on each image in a different color (magenta), to increase the contrast with the background, and mean images for each treatment were generated.

The diets were analyzed for dry matter and crude protein according to AOAC [37] and total lipid fraction according to Folch et al. [38]. Total carotenoids concentration in the test diets was determined following the protocol of Parisenti et al. [39]. Under low-light conditions, total carotenoids were extracted from 1 g of homogenized feed sample with 15 mL of acetone and shacked. Each extract was collected in separating tubes. A volume of 12.5 mL of hexane, 50 mL of water and 0.25 g of NaCl were added for separating water-soluble compounds. Tubes were kept in dark for 20 min and shaken once. Hexane extract was filtered and dehydrated by anhydrous sodium sulphate and poured in a volumetric tube (25 mL). Total carotenoid concentration was determined spectrophotometrically (spectrophotometer Hewlett-Packard, HP 8452A; Cheadle Heath, Stockport Cheshire, UK) at 470 nm and calculated according to a standard curve of astaxanthin (Sigma, St. Louis, MO, USA, 98% purity). Pigment concentration in experimental diets was expressed in mmkg-1 on dry weight basis.

Differences in length and weight measurements between treatments were tested by means of one-way ANOVA with Tuckey’s post hoc test for means comparison. Differences in the number of yellow pixels on lateral and frontal profile among treatments and replicates of each treatment were tested by means of a two-way Analysis of Similarities (ANOSIM [40]) with post-hoc pairwise comparison probabilities (Bonferroni corrected). Data were standardized according to the total number of pixels for each individual and Manhattan distance was used; the number of permutations was set to 999. The presence of a significant relationship between total carotenoid concentration in the diet and number of yellow pixels (in frontal and lateral side) was investigated by means of the Mantel test [41] a non-parametrical test of correlation between two matrices based on permutations. Manhattan and Euclidean distances were selected as measures of distance, while the Spearman’s correlation coefficient was used to calculate the R value. Number of permutations was set to 9999.

All analyses were carried out with R 3.9.9 software (Foundation for Statistical Computing, Vienna, Austria).

All procedures involving fish manipulation were carried out in accordance with the EU legal framework relating to the protection of animals used for scientific purposes (Directive 2010/63/EU). They were approved by the Animal Welfare Committee of the University of Udine (OPBA) and authorized by the Italian Ministry of Health (permission n. 290/2019-PR).

## 3. Results and Discussion

### 3.1. General Results

At the end of the trial, the dietary treatments resulted in significant changes in fish total length (F = 20.5; *p* < 0.001) and final weight (F = 5.9; *p* < 0.001). Significant differences among dietary treatments are reported in Table 3. Seabreams fed diets MA10 and CV grew less in weight than those fed diets H40, P20 and P40 which did not differ from each other, while the other diets gave rise to intermediate values. As far as total length was concerned, fish fed CV, H10P30 and MA10 diets grew significantly less than those fed diets H20, H40, P20 and P40, while the other diets gave rise to intermediate values.

### 3.2. Skin Pigmentation

To date, skin color pattern in Sparidae has generally been measured in specific body areas by means of a colorimeter or using human visual scoring (Table 1). The application of image analysis tools to the study of skin pigmentation has the double advantage of offering more objective measurements under uniform conditions than other traditional methods and of returning results on the overall pigmentation pattern. Results of color analysis carried out in this study are summarized in Table 3. Number of yellow pixels detected was on average higher in frontal images (8033 ± 271) than in lateral ones (3836 ± 105), but they were less dispersed (SDD frontal: 80.7 ± 0.7; SDD lateral: 148.7 ± 1.2) and concentrated in the area between the eyes.

**Figure 1:**
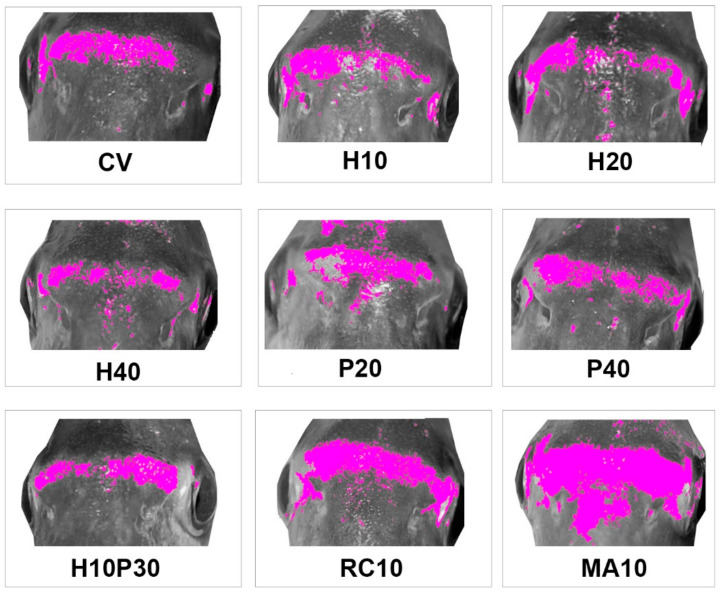
Average frontal profile patterns of pigmentation in gilthead seabream fed with experimental diets. Mean images for each treatment. Yellow pixels have been plotted in magenta to be more evident. CV: vegetable control; H: *Hermetia illucens*; P: poultry by-product; RC: red swamp crayfish; MA: microalgae dried biomass.

**Figure 2:**
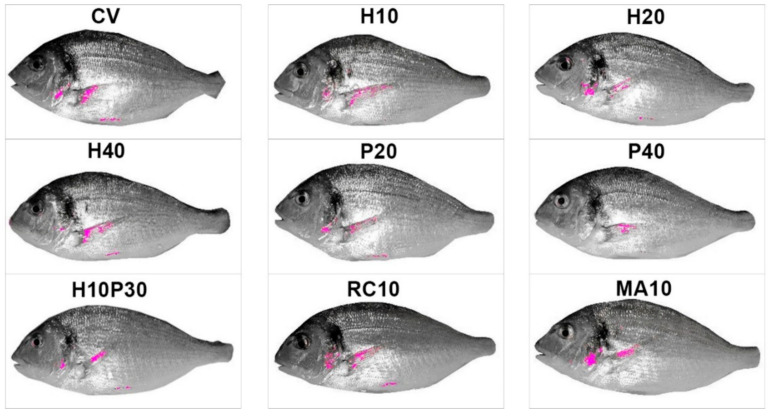
Average lateral profile patterns of pigmentation in gilthead seabream fed with experimental diets. Mean images for each treatment. Yellow pixels have been plotted in magenta to be more evident. CV: vegetable control; H: *Hermetia illucens*; P: poultry by-product; RC: red swamp crayfish; MA: microalgae dried biomass.

**Figure 3:**
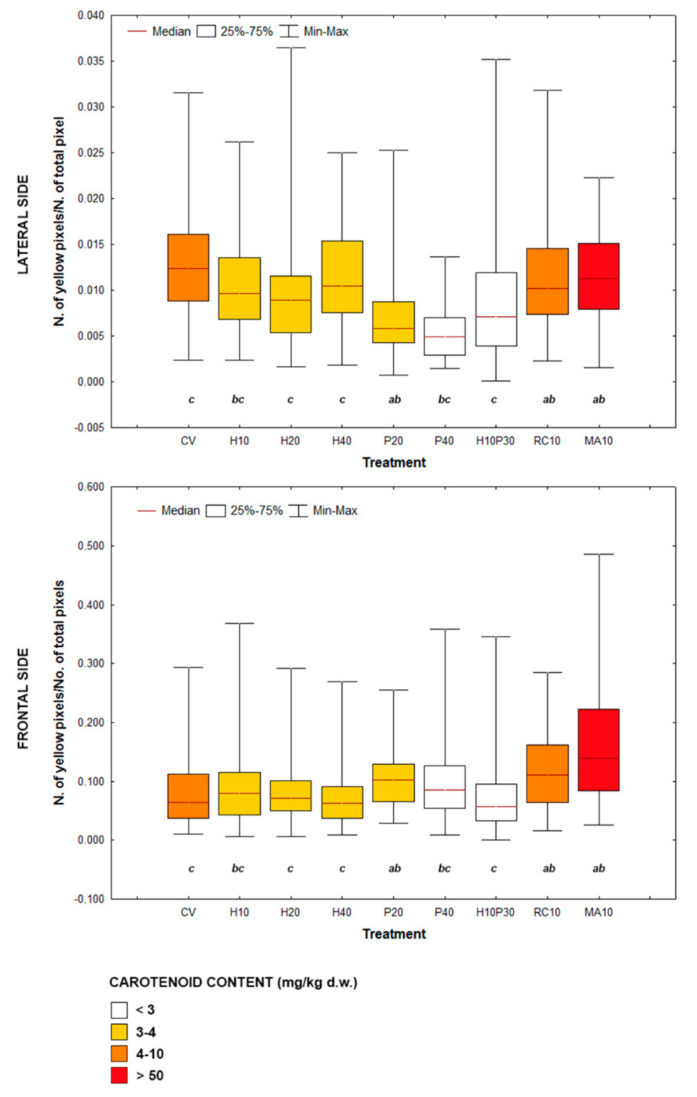
Box plots showing variability of the number of yellow pixels on lateral and frontal sides of experimental groups. Different letters indicate significant differences among treatments (ANOSIM; *p* < 0.05); similar letters depict no significant differences. Color shade is a measure of total carotenoids concentration in the diet. CV: vegetable control; H: *Hermetia illucens*; P: poultry by-product; RC: red swamp crayfish; MA: microalgae dried biomass.

Fish from different treatments showed yellow areas with different dimensions and pixel density on the frontal profile (Figure 1). The experimental groups showing the most intense and widespread yellow skin pigmentation were MA10 and RC10, followed by P20 and CV. Lateral profile was characterized by different patterns of pigmentation (Figure 2): (i) absence or very limited yellow areas, as in P40; (ii) presence of two or three small yellow areas on the operculum, on the pectoral fin and on the belly (H10, H20, H40, P20 and H10P30); (iii) presence of two or three well defined yellow areas on the operculum, on the pectoral fin and on the belly (CV, MA10 and RC10). Skin pigmentation, measured as number of yellow pixels in lateral and frontal profile, was significantly different among treatments (R = 44.09; *p* < 0.001), but not among replicates (Figure 3).

From ANOSIM post-hoc comparisons, fish fed microalgae, crayfish and poultry by-product in the lowest percentage (P20) clustered together, but while MA10 and RC10 showed similar pattern of pigmentation of frontal and lateral sides, P20 was poorly colored on the lateral side and showed an intermediate yellowish coloration intensity on the forefront. The vegetable control was intermediate between the MA10, RC10 and P20 groups and the H20, H40 and H10P30 groups, and was pigmented mainly on the lateral side. Seabream fed insects (H10, H20, H40) showed a poor yellow pigmentation on the lateral side, while a gradient was evident on the forefront, with at lower levels of substitution of vegetable ingredients with *H. illucens* meal corresponded a higher number of yellow pixels.

The SDD was significantly different both among treatments and among replicates, even if with low *r^2^* values: *r^2^*_treat_ = 0.05, *p* < 0.01; *r^2^*_rep_ = 0.01, *p* < 0.05 (ANOSIM). Therefore, no clear indications can be drawn from these data, which show very high intra-treatment variability. On average, lateral SDD was higher than frontal one, the lowest in H10 (145.2 ± 1.2), the highest in P40 (153.6 ± 0.6), while the dispersion of points on frontal profile was on average the highest in H10P30 (87.1 ± 7.9) and the lowest in H20 (73.1 ± 7.2). In this study, the yellowish pigmentation in lateral and frontal areas, expressed as number of yellow pixels, was positively correlated with the total carotenoid concentration in the diet (R = 0.38; *p* < 0.001).

All test diets in this study contained vegetable ingredients, such as cereal glutens, that are good sources of yellow carotenoids [42] and may easily explain the notably good skin pigmentation pattern here observed in fish fed diet CV. Diets including graded levels of the black soldier fly larvae or poultry by-product meals singly or in association, to replace plant protein-rich ingredients, resulted in diluted total carotenoid content relative to CV diet, which fits with a slightly reduced yellow pixel number here observed mostly in the opercular region of fish fed those diets. This is apparently consistent with existing data on the carotenoids content in the black soldier fly larvae which ranged between 2.00–2.15 mg kg^−1^ [43,44], much lower than in cereal glutens. Although no data have been found for poultry by-product meal, it is assumed that it contains low levels of carotenoids (1.83 mg kg^−1^ [45]). No studies are available on the effects of diets including such processed animal proteins on fish skin pigmentation. Fish fed the diet including a blend of two microalgae *T. lutea* and *T. suecica* dried biomass at 11% in weight (MA10) was definitively the one showing the highest number of yellow pixels as a consequence of a notably high content of carotenoids in the corresponding diet (Table 2, Figure 3). Both microalgae are known to be rich in carotenoids, mainly fucoxanthin for *T. lutea* and lutein for *T. suecica* [46], and the same blend of the two microalgae or *T. lutea* alone, when included at various levels in the diet, have recently been found to increase redness in both dorsal and ventral regions of the skin in the European seabass (*D. labrax*) [47,48]. Improved pigmentation of seabream with the diet MA10 in this study is not surprising, since many studies showed similar effects in red tilapia (*Oreochromis* spp.) and ornamental carp (*Cyprinus carpio*) fed diets containing dried biomass of other microalgae such as *Arthrospira platensis* [49,50], and in gilthead seabream fed diets supplemented with the carotenoid-rich *H. pluvialis* [17,51]. Astaxanthin from *H. pluvialis* was also tested in diets for *P. pagrus* alevins, giving their skin an acceptable pink color, compared to the grayish one of farmed fish fed with diets not supplemented with carotenoids [22]. The green alga *Chlorella vulgaris* was also added to a basal diet for *S. aurata*, determining a significant carotenoids deposition in four skin zones (i.e., forefront, operculum, dorsal fin, abdominal area), but not in the muscle [18]. Diets implemented with 2.5% of the marine diatom *Phaeodactylum tricornutum* did not impact organoleptic properties of *S. aurata* fillets [26] but resulted in a significantly more vivid yellow pigmentation of the operculum.

In this study, skin pigmentation pattern in fish fed the diet including a meal obtained from red swamp crayfish was very similar to that attained by those fed diet MA10. Processed products derived from *P. clarkii* have been already used as a source of pigments in fish diets [23] and as a source of chitin in feeds for shrimps [52]. Astaxanthin concentration measured in the experimental crayfish meal used for the RC10 diet was 11.95 mg 100g^−1^, consistent with values found by Pérez-Gálvez et al. [53] and may account for improved skin pigmentation. The effects of crustacean meal inclusion in aquafeeds on Sparidae skin pigmentation have been poorly investigated to date. Including *Plesionika* sp. meal at 12% by weight in a commercial diet did not affect growth and survival of red porgy alevins but resulted in an intense pink color of the skin like that of wild fish [21]. Including red swamp crayfish meal in diets for red porgy resulted in enhanced skin redness and yellowness relative to that exhibited by fish given a control fishmeal-based diet [23]. Red porgy alevins fed diets supplemented with marine spider crab *Paramola cuvieri* showed significantly elevated skin redness and carotenoids concentration in skin samples [25].

## 4. Conclusions

Farmed gilthead seabream often exhibit gray pigmentation, different from the golden yellow coloration on forefront and operculum of their wild counterparts; thus, commercial feeds are often supplemented with carotenoids to enhance skin pigmentation and increase consumer acceptance of farmed products. The present study, through an automatic and non-invasive image analysis tool, has shown that the pattern of yellowish pigmentation of the skin in gilthead seabream could be affected by varying nature and levels of major protein sources in diets deprived of fish meal and already including vegetable protein-rich ingredients. Replacing graded levels of vegetable ingredients rich in carotenoids with defatted *H. illucens* pupae or poultry by product meals had minor impact on skin pigmentation possibly due to a marginal contribution of carotenoid supplied by this ingredient. The inclusion of a mix of dried microalgae *T. suecica* and *T. lutea* and red swamp crayfish meal had a beneficial effect on *S. aurata* skin pigmentation, making these ingredients candidates as potentially effective natural sources of carotenoids. Mechanisms by which pigments from these sources are made available for storage in fish tegument should be studied in order to optimize their levels of inclusion in fish diets [54].

## Figures and Tables

**Table 1:** Effects of diet on skin pigmentation in Sparidae—available literature.

Species	Feed Ingredient	Main Results	Reference
*Chrisophrys major*	*Euphasia superba*; *Neomysis* sp.	Higher rate of carotenoid deposition and distinct pigmentation	[20]
*Sparus aurata*	*Haematococcus pluvialis*; synthetic astaxanthin	Total carotenoid concentration of the skin was not significantly affected by the dietary pigment sources	[17]
*Sparus aurata*	*Chorella vulgaris*; synthetic astaxanthin	Carotenoids content in muscle was very low; no variation in the amount of carotenoid in the skin tissue	[18]
*Pagrus pagrus*	*Plesionika* sp.	Pink-colored skin like that of the wild fish	[21]
*Pagrus auratus*	Synthethic astaxanthin (36 or 72 mg/kg)	Diet containing 72 mg astaxanthin/kg gave more reddish coloration; color saturation after six weeks	[10]
*Pagrus pagrus*	Astaxathin; β-carotene; lycopene	Astaxanthin increased skin carotenoid content; β-carotene and lycopene had no effect	[3]
*Pagrus pagrus*	Synthetic canthaxanthin; astaxanthin from shrimp shell	Only astaxanthin from shrimp shell meal gave skin an overall natural reddish coloration	[5]
*Pagrus pagrus*	*Haematococcus pluvialis*; synthesized astaxanthin	Synthetic and natural astaxanthin increased pink skin pigmentation; esterified astaxanthin gave better results in terms of skin pigmentation	[22]
*Pagrus auratus*	Astaxanthin; canthaxanthin; apocarotenoic acid ethyl ester; selected combinations of the above	Astaxanthin conferred greatest skin pigmentation	[11]
*Sparus aurata*	*Capsicum annuum*; *Daucus carota*	Carotenoid supplementation from red pepper meal significantly increased skin carotenoids content; gilthead seabream failed to use carotenoids in carrot	[19]
*Pagrus pagrus*	*Procambarus clarkii* meal; *Chaceon affinis* meal	*P. clarkii* meal was shown to be a more efficient pigment source for this species	[23]
*Pagrus pagrus*	Astaxanthin; xanthophylls	Supplementation of synthetic carotenoids affected skin color indexes: higher values of redness, yellowness, and chroma recorded in the fish fed with astaxanthin	[24]
*Pagrus pagrus*	*Paramola cuvieri*; *Diadema africanum*	*P. cuvieri* meal inclusion improved skin colouration; *D. africanum* meal promoted yellow coloration	[25]
*Sparus aurata*	*Phaeodactylum tricornutum*	*P. tricornutum* biomass inclusion induced a more vivid yellow coloration of operculum	[26]

**Table 2:** Ingredient composition, crude protein, lipid levels and total carotenoids content of the test diets. CV: vegetable control; H: Hermetia illucens; P: poultry by-product; RC: red swamp crayfish; MA: microalgae dried biomass.

Test Diets									
Ingredients	CV	H10	H20	H40	P20	P40	H10P30	RC10	MA10
Ingredient composition%									
Plant-protein mix ^1^	69.0	60.5	52.6	36.6	52.5	35.4	35.4	58.8	58.3
*Hermetia* pupae meal ^2^	-	8.1	16.2	32.4	-	-	8.1	-	-
PBM ^3^	-	-	-	-	13.8	27.5	20.6	-	-
RCM ^4^	-	-	-	-	-	-	-	10.1	-
MA mix ^5^	-	-	-	-	-	-	-	-	11.6
Feeding stimulants ^6^	5.5	5.5	5.5	5.5	5.5	5.5	5.5	5.5	5.5
Fish oil	6.2	6.2	6.2	6.2	6.2	6.2	6.2	6.2	6.2
Veg oil mix ^7^	11.4	10.0	8.4	5.4	9.8	8.2	7.4	10.8	10.5
Wheat meal	0.4	0.6	1.6	4.5	3.0	5.6	5.5	0.4	
Whole pea	3.0	4.8	5.8	6.0	6.2	9.0	8.8	4.1	4.0
Vitamin, mineral suppl. ^8^	3.5	3.4	3.1	2.9	2.6	2.2	2.1	3.4	3.2
L-Lys	0.5	0.5	0.2	0.2	0.1	0.1	0.1	0.3	0.3
DL-Met	0.5	0.4	0.4	0.3	0.3	0.3	0.3	0.4	0.4
Proximate composition as fed (%)									
Crude protein	44.9	45.0	44.9	45.2	45.2	45.1	45.1	44.9	45.0
Crude fat	20.1	20.0	20.1	20.1	20.3	20.2	19.9	19.9	20.1
Carotenoids concentration (mg kg^−1^ d.w.)	4.6	3.5	3.5	3.5	3.3	2.4	2.7	5.4	234.2

^1^ Plant-protein sources mix. % composition: dehulled, toasted soybean meal, 39; soy protein concentrate—Soycomil, 20; maize gluten, 18; wheat gluten, 15, rapeseed meal, 8. ^2^ Protein X from PROTIX, The Netherlands. ^3^ Poultry by-product meal from Azienda Agricola Tre Valli; Verona, Italy. ^4^ RC (red swamp crayfish meal) from CREA- Animal Husbandry and Aquaculture ^5^ Microalgae dried biomass mixture % composition: *Tisochrysis lutea* F&M-M36, 64; *Tetraselmis sueica*, 36. Supplied by University of Florence, Italy.^6^ Feeding stimulants g/100 diet: CPSP90—Sopropeche, France, 3.5; Squid meal, 2.0. ^7^ Vegetable oil mixture % composition: rapeseed oil, 56; linseed oil, 26; palm oil, 18. ^8.^ Supplying per kg of vitamin supplement: Vit. A, 4,000,000 IU; Vit D3, 850,000 IU; Vit. K3, 5000 mg; Vit.B1, 4000 mg; Vit. B2, 10,000 mg: Vit B3, 15,000 mg; Vit. B5, 35,000 mg; Vit B6, 5000 mg, Vit. B9, 3000 mg; Vit. B12, 50 mg, Biotin, 350 mg; Choline, 600 mg; Inositol, 150,000 mg. Supplying per kg of mineral supplement: Ca, 77,000 mg; Cu, 2500 mg; Fe, 30,000 mg; I, 750 mg; Se, 10,000 mg; Zn, 25 mg. Wherever not specified, the ingredients composing the diets were obtained from local providers.

**Table 3:** Number of specimens (N), total length (TL), weight (W), number of lateral and frontal yellow pixels, dispersion index of lateral and frontal yellow pixels, percentage of lateral and frontal yellow pixels on the total pixel number of the image. Values are reported as the treatment mean ± S.D. Different letters (a,b,c,d) indicate significant differences among treatments (Tukey test; *p* < 0.05); similar letters depict no significant differences. CV: vegetable control; H: *Hermetia illucens*; P: poultry by-product; RC: red swamp crayfish; MA: microalgae dried biomass.

Treatment	*N*	TL (cm)	W (g)	Lateral Pixels	Lateral SDD	Lateral%	Frontal Pixels	Frontal SDD	Frontal %
CV	55	21.7 ± 1.0 ^a^	177.7 ± 22.4 ^ab^	4641 ± 327	147.5 ± 1.3	1.25 ± 0.08	6560 ± 664	76.8 ± 7.2	8.20 ± 0.83
H10	53	22.5 ± 1.0 ^cd^	186.8 ± 26.9 ^bc^	4042 ± 255	145.2 ± 1.2	1.09 ± 0.0.8	6956 ± 684	86.7 ± 7.6	8.69 ± 0.86
H20	54	23.0 ± 0.8 ^bd^	187.5 ± 22.0 ^bc^	4052 ± 376	152.8 ± 1.1	1.00 ± 0.0.9	6534 ± 529	73.1 ± 7.2	8.17 ± 0.66
H40	53	23.3 ± 1.0 ^bd^	192.2 ± 22.2 ^c^	4506 ± 303	150.1 ± 1.2	1.12 ± 0.07	6003 ± 619	81.2 ± 6.9	7.50 ± 0.77
P20	53	22.9 ± 0.9 ^bd^	191.6 ± 24.3 ^c^	2757 ± 221	148.6 ± 0.7	0.68 ± 0.06	8717 ± 619	78.6 ± 10.6	10.89 ± 0.90
P40	52	23.0 ± 0.8 ^bd^	192.3 ± 23.2 ^c^	2211 ± 191	153.6 ± 0.6	0.54 ± 0.04	7795 ± 679	82.1 ± 7.8	9.74 ± 0.85
H10P30	53	22.0 ± 0.8 ^ac^	190.7 ± 21.6 ^bc^	3214 ± 332	146.3 ± 0.9	0.85 ± 0.09	6386 ± 804	87.1 ± 7.9	7.98 ± 1.00
RC10	54	22.3 ± 0.8 ^c^	180.5 ± 23.9 ^abc^	4501 ± 335	147.1 ± 1.2	1.17 ± 0.07	9698 ± 722	79.9 ± 9.1	12.12 ± 1.57
MA10	54	22.2 ± 0.8 ^ac^	166.9 ± 19.8 ^a^	4517 ± 279	146.8 ± 1.3	1.16 ± 0.09	13,613 ± 1,255	81.1 ± 14.2	17.01 ± 0.77
